# F-Box Family Genes, *LTSF1* and *LTSF2,* Regulate Low-Temperature Stress Tolerance in Pepper (*Capsicum chinense*)

**DOI:** 10.3390/plants9091186

**Published:** 2020-09-11

**Authors:** Jelli Venkatesh, Min-Young Kang, Li Liu, Jin-Kyung Kwon, Byoung-Cheorl Kang

**Affiliations:** 1 Department of Agriculture, Forestry and Bioresources, Research Institute of Agriculture and Life Sciences, Plant Genomics and Breeding Institute, College of Agriculture and Life Sciences, Seoul National University, Seoul 08826, Korea; jvs15@snu.ac.kr (J.V.); kmyjj3802@korea.kr (M.-Y.K.); kwonjk@snu.ac.kr (J.-K.K.); 2 Global Institute for Food Security, University of Saskatchewan, Saskatoon, SK S7N 5A8, Canada; li.liu-green@gifs.ca

**Keywords:** F-box protein, low-temperature stress, proteasomal degradation, transgenics, virus-induced gene silencing, SCF complex

## Abstract

The F-box proteins belong to a family of regulatory proteins that play key roles in the proteasomal degradation of other proteins. Plant F-box proteins are functionally diverse, and the precise roles of many such proteins in growth and development are not known. Previously, two low-temperature-sensitive F-box protein family genes (*LTSF1* and *LTSF2*) were identified as candidates responsible for the sensitivity to low temperatures in the pepper (*Capsicum chinense*) cultivar ‘*sy-2*’. In the present study, we showed that the virus-induced gene silencing of these genes stunted plant growth and caused abnormal leaf development under low-temperature conditions, similar to what was observed in the low-temperature-sensitive ‘*sy-2*’ line. Protein–protein interaction analyses revealed that the LTSF1 and LTSF2 proteins interacted with S-phase kinase-associated protein 1 (SKP1), part of the Skp, Cullin, F-box-containing (SCF) complex that catalyzes the ubiquitination of proteins for degradation, suggesting a role for LTSF1 and LTSF2 in protein degradation. Furthermore, transgenic *Nicotiana benthamiana* plants overexpressing the pepper *LTSF1* gene showed an increased tolerance to low-temperature stress and a higher expression of the genes encoding antioxidant enzymes. Taken together, these results suggest that the LTSF1 and LTSF2 F-box proteins are a functional component of the SCF complex and may positively regulate low-temperature stress tolerance by activating antioxidant-enzyme activities.

## 1. Introduction

In plants, low temperatures affect many physiological processes, including water and nutrient uptake, photosynthesis, immune responses, growth, and development, as well as the geographical distribution of plants [1,2]. Enhancing the low-temperature tolerance of temperature-sensitive crops is necessary to increase their productivity [3,4,5]. Pepper, an important fruit crop grown worldwide, is temperature-sensitive, with an optimum temperature of 25–30 °C [6,7]. Deviations from these temperatures can adversely affect growth and development, resulting in a variety of developmental and physiological disorders [6,7,8].

Many plant species overcome the adverse effects of low and non-freezing temperatures through complex adaptive mechanisms which are associated with the extensive reprogramming of the expression of a number of cold-responsive genes and subsequent changes in numerous physiological and biochemical processes, such as membrane stability, calcium ion fluxes, and changes in the properties of the cell wall and the plasma membrane [9,10,11,12]. To elucidate the molecular basis of low-temperature sensitivity in plants, extensive studies have been performed using low-temperature-sensitive mutants. The *Arabidopsis thaliana fatty acid desaturase 2* (*fad2*) mutant, which is deficient in the endoplasmic reticulum 18:1 desaturase, displays abnormal leaves and a dwarf phenotype under low temperatures [13,14]. The *Arabidopsis nonphotochemical quenching 1* (*npq1*) mutant, defective in xanthophyll biosynthesis, excessively accumulates reactive oxygen species (ROS) [15]. The *Arabidopsis BONZAI 1* (*BON1*) and *BON1*-*associated protein* (*BAP1*) genes were suggested to function directly in the regulation of cell expansion and cell division at lower temperatures, although the miniature *bon1* mutant plants were fertile [16]. The maize (*Zea mays*) mutant inbred line *M11* and the rice (*Oryza sativa*) *low temperature albino 1* (*lta1*) mutants contained remarkably low levels of chlorophyll [17,18]. Despite these insights, little is known about the genes and mechanisms regulating low-temperature tolerance in plants.

The pepper cultivar ‘*sy-2*’ is a temperature-sensitive landrace local to the Seychelles, and shows developmental defects, such as abnormal leaf growth, chlorophyll deficiency, the excessive accumulation of ROS, and altered fatty acid biosynthesis, when grown at temperatures lower than 24 °C [6,8,19]. Using genetic mapping, two low-temperature-sensitive F-box (LTSF) proteins, LTSF1 and LTSF2 (encoded by *ORF10* and *ORF20*, respectively), belonging to the Skp, Cullin, F-box-containing (SCF) complex of ubiquitin-proteasome machinery, were identified as candidates responsible for the low-temperature sensitivity in the pepper cultivar ‘*sy-2*’ [19]; however, the functional relevance of the *LTSF1* and *LTSF2* genes in the temperature sensitivity of the ‘*sy-2*’ cultivar is still unclear.

The ubiquitin-proteasome pathway plays a central regulatory role in the selective degradation of target proteins through a series of steps. First, the ubiquitin is activated by the E1 enzyme (ubiquitin-activating enzyme), then the activated ubiquitin is transferred to E2 (ubiquitin-conjugating enzyme). The E2 transfers ubiquitin to the E3 (ubiquitin protein ligase) after which the ubiquitin is transferred to the target protein by the E3 complex [20]. F-box proteins are constituent subunits of the multi-protein E3 ubiquitin ligase (also known as the SCF complex) and confer target specificity during the 26S proteasome-mediated protein degradation [20]. The F-box family is one of the largest multigene families in plants [21]. A large number of F-box proteins have been identified in searches for their highly-conserved N-terminal F-box domain, and a total of 694, 687, 337, and 156 F-box family genes have been reported in *Arabidopsis*, rice, poplar, and grape, respectively [22,23]. F-box protein families can be grouped into several subfamilies based on their C-terminal target-specific protein–protein interaction domains, such as the leucine-rich repeats (LRRs), tubby (TUB), tetratricopeptide (TPR), kelch repeats, Trp-Asp forty-amino-acid repeat (WD-40), armadillo repeats (ARMs), and RING finger domains [24,25,26]. The presence of numerous diverse F-box proteins facilitates the formation of diverse SCF complexes that can recognize a broad array of substrates, and can, thus, be associated with the regulation of numerous biophysiological processes, such as plant growth and development, hormonal responses, biotic and abiotic stress responses, and senescence [21,27].

In this study, we performed a functional characterization of the low-temperature sensitive pepper F-box protein genes *LTSF1* and *LTSF2* using virus-induced gene silencing (VIGS) and transgenic approaches. The silencing of *LTSF1* and *LTSF2* expression in the low-temperature-tolerant pepper line ‘No.3341’ resulted in a low-temperature-sensitive phenotype similar to ‘*sy-2*’. Transgenic tobacco (*Nicotiana benthamiana*) lines overexpressing the pepper *LTSF1* allele from ‘No.3341’ displayed an enhanced tolerance to low temperatures relative to the control.

## 2. Results

### 2.1. LTSF1 and LTSF2 Sequence Analysis

**Figure 1:**
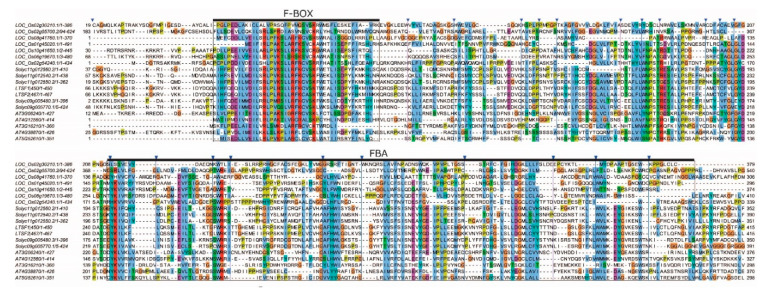
Multiple-sequence alignments of two low-temperature-sensitive F-box (LTSF) proteins, LTSF1 and LTSF2, in pepper (‘No.3341’) with their homologs in *Arabidopsis*, tomato, and rice. The predicted F-box and F-box-associated (FBA) domains are indicated above the alignment. Amino acid (AA) residues are colored according to ClustalX color code using Jalview tool.

We characterized the *LTSF1* and *LTSF2* genes and proteins believed to be affected in the ‘*sy-2*’ pepper mutant [19]. The predicted LTSF1 and LTSF2 proteins consisted of 450 and 467 amino acids (AAs), respectively (Supplementary Materials Figure S1), with predicted molecular weights of about 51.8 and isoelectric points of 9.31. A search using the InterPro protein sequence analysis and classification software tool identified two conserved domains in both LTSF1 and LTSF2: an F-box domain (*IPR001810*) in the N-terminus and an F-box-associated (FBA) interaction motif (*IPR017451*) in the C-terminus (Figure 1). The presence of the F-box domain indicated that LTSF1 and LTSF2 are F-box proteins, which bind to S-phase kinase-associated protein 1 (SKP1) to form the SCF complex. FBA motifs are generally involved in interactions with the target proteins to be ubiquitinated for proteasomal degradation.

**Figure 2:**
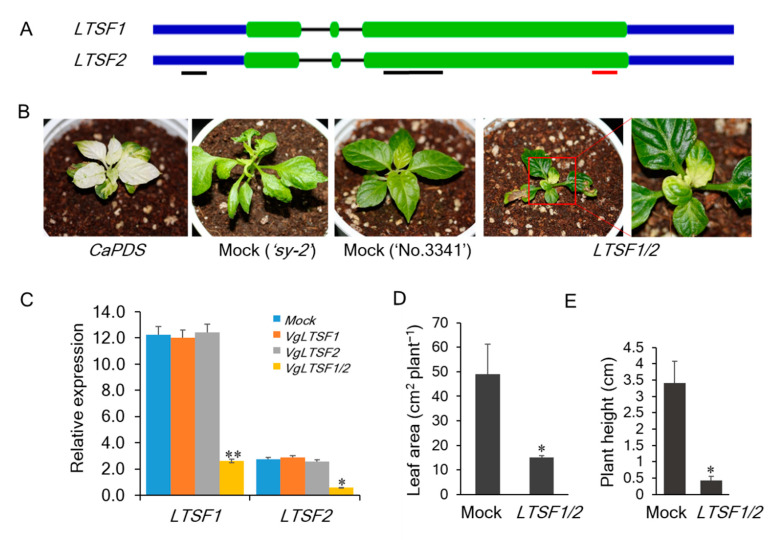
Virus-induced gene silencing (VIGS) of the pepper *LTSF1* and *LTSF2* genes in wild-type (‘No.3341’) plants. (**A**) Schematic structure of the *LTSF1* and *LTSF2* gene sequences. Closed green boxes and solid black lines indicate exons and introns, respectively. Blue lines indicate untranslated regions (UTRs). The VIGS target sites in *LTSF1* and *LTSF2* are underlined with black lines and qPCR target region is underlined with red line. (**B**) VIGS pepper phenotypes. Pepper plants with VIGS of *Capsicum phytoene desaturase* (*CaPDS*) showed a photobleaching phenotype. The double silencing of the *LTSF1/2* genes resulted in plants with an abnormal phenotype. The rightmost image is a magnification of the area highlighted by a red square. (**C**) Quantitative PCR (qPCR) analysis of plants in which *LTSF1* and/or *LTSF2* were silenced using VIGS (*Vg*). *VgLTSF1* and *VgLTSF2* refer to the VIGS constructs targeting the UTRs. (**D**) Quantitative analysis of leaf area in mock (‘No.3341’) and *LTSF1/2* silenced plants. (**E**) Quantitative analysis of plant height in mock (‘No.3341’) and *LTSF1/2* silenced plants. The error bars indicate the mean ± standard deviation (SD) of six biological replicates. * *p* < 0.05 and ** *p* < 0.01 vs. control (mock).

The LTSF1 and LTSF2 proteins share a 91% sequence similarity, with a single AA difference in their F-box domains. The protein sequence alignment of the F-box domains of LTSF1 and LTSF2 in pepper and other plant species, including Arabidopsis, rice, and tomato, revealed a high sequence conservation in the F-box domain (Figure 1). LTSF1 and LTSF2 showed a maximum sequence identity of 41–42% with the tomato F-box protein Solyc09g005480, and shared a sequence identity of 24–25% with the FBA motif-containing Arabidopsis At4g12560 protein (CPR1; CONSTITUTIVE EXPRESSER OF PR GENES 1); At3g06240 (a F-box/kelch-repeat protein); and rice Os02g54240, a stress-responsive F-box protein. A comparison of the genomic structures revealed that both the *LTSF1* and *LTSF2* genes contain two introns (Figure 2A).

### 2.2. VIGS Analysis

To test the functional roles of *LTSF1* and *LTSF2* in low-temperature sensitivity, we silenced their expression in wild-type pepper (‘No.3341’) using *tobacco rattle virus* (*TRV*)-mediated VIGS (Figure 2A,B). VIGS vectors were constructed targeting the UTR or coding (CDS) regions. To confirm the efficient silencing of the genes, a qPCR was performed in the silenced plants. No significant changes were observed in the expression levels of the *LTSF1* and *LTSF2* genes in plants infiltrated with constructs targeting either *LTSF1* or *LTSF2* gene UTR regions due to the unsuccessful gene silencing, possibly due to shorter VIGS target region of 135 bp and 127 bp, respectively for *LTSF1* and *LTSF2.* However, in plants infiltrated with VIGS construct (*LTSF1/2-*double-silenced plants) targeting CDS regions of both *LTSF1* and *LTSF2* with 305 bp target length (Figure 2A), the expression levels of these genes were approximately four times lower compared with the mock plants (Figure 2C). The double silencing of the *LTSF1* and *LTSF2* genes by targeting their CDS region resulted in an abnormal phenotype and severe growth retardation under low-temperature conditions (20 °C), consistent with the suppressed expression of *LTSF1* and *LTSF2* in these plants (Figure 2B,C). The *LTSF1*/*2*-silenced plants produced small, irregularly shaped, pale, and thick leaves, similar to the phenotype observed in the ‘*sy-2*’ pepper mutant, which exhibits thick irregularly shaped leaves when grown under low-temperature conditions [19]. Plant height and leaf area were markedly affected in the *LTSF1*/*2*-silenced plants as compared with mock plants infiltrated with empty vector (TRV2:00). These results indicate an important role for the F-box genes in low-temperature sensitivity and morphogenesis in the pepper plants.

### 2.3. LTSF1 and LTSF2 Interaction with SKP1

**Figure 3:**
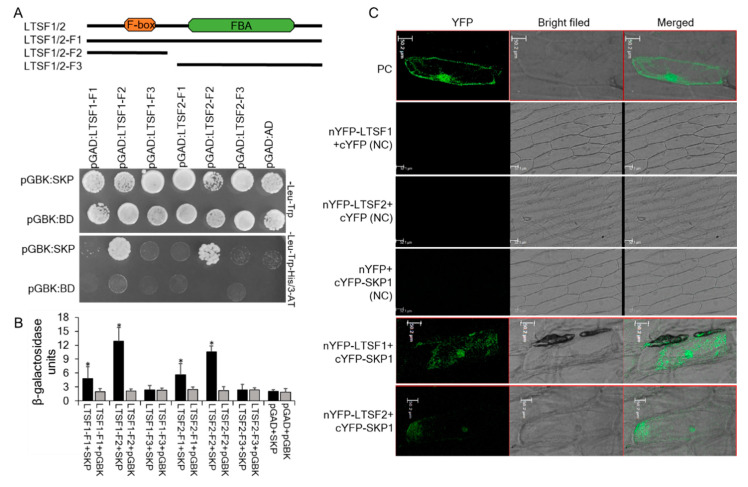
Protein–protein interaction studies involving LTSF1 and LTSF2. (**A**) Yeast two-hybrid assays analyzing the interactions between LTSF1/2 and SKP1. The LTSF1, LTSF2, and SKP1 coding sequences were cloned into the pGAD-T7 and pGBK-T7 vectors. Yeast AH109 cells transformed with the designated plasmid combinations were grown on SD/-Leu/-Trp and SD/-Leu/-Trp/-His/+3-aminotriazol (3-AT) media. (**B**) β-galactosidase (GUS) activity assay. Error bars indicate SD of five biological replicates of relative GUS activity assay. The asterisk indicates a significant difference compared with the negative construct (*p* < 0.05). (**C**) Bimolecular fluorescence complementation assay of the interactions between pepper LTSF1/2 and SKP1. The LTSF1, LTSF2, and SKP1 coding sequences were cloned into the pSPYCE and pSPYNE vectors. The designated plasmid combinations were co-bombarded into onion epidermal cells for the transient expression of yellow fluorescent protein (YFP), and the fluorescence signals were visualized using a laser-scanning confocal microscope. PC and NC indicate positive and negative controls.

*LTSF1* and *LTSF2* were predicted to encode F-box proteins containing a C-terminal FBA domain (Figure 1). To determine whether the LTSF1 and LTSF2 proteins are functional components of the SCF complex, protein–protein interaction studies were carried out (Figure 3A). SKP1 interacts with F-box proteins by binding to their conserved F-box domain [20]; therefore, we performed yeast two-hybrid (Y2H) assays to assess whether pepper SKP1 can interact with the Sy-2 candidates LTSF1 and LTSF2. No significant growth was detected in yeast cells carrying the control vectors on the selection medium (SD/-Leu/-Trp/-His/3-AT); however, the yeast cells carrying the pGAD-T7 vector containing the F-box domain sequence of *LTSF1* or *LTSF2* (*LTSF1*/2*-F2*) and pGBK-T7:SKP1 grew well (Figure 3A), suggesting that LTSF1/LTSF2 can interact with SKP1. Consistent with these results, β-galactosidase (GUS) assay raveled a strong interaction between LTSF1/LTSF2 and SKP1 (Figure 3B). The growth of the yeast cells carrying the pGAD-T7 vector with the full-length sequence of *LTSF1* or *LTSF2* (*LTSF1*/2*-F1*) and the pGBK-T7:SKP1 was inhibited on the SD plate, however (Figure 3A). This weak or absent interaction between the full-length LTSF1/LTSF2 proteins and SKP1 may suggest that the C-terminal FBA domains may have an inhibitory effect in yeast cells. Alternatively, it is possible that the low effectivity of interaction could also be caused by different expression levels of the proteins.

To confirm the protein–protein interaction between LTSF1/LTSF2 and SKP1 in plant cells, a bimolecular fluorescence complementation (BiFC) assay was performed. LTSF1 and LTSF2 were fused with the C-terminus of YFP, and SKP1 was fused with the N-terminus of YFP. These constructs were then co-bombarded into onion epidermal cells. The negative control failed to produce a BiFC signal, while a strong fluorescent signal was observed throughout the cells transformed with the positive controls. A strong fluorescent signal was observed in the nuclei, cytoplasm, and cell membranes of epidermal cells co-bombarded with the YFP fusion proteins of LTSF1 and SKP1 (Figure 3C), indicating a strong interaction between LTSF1 and SKP1, whereas a weak fluorescent signal was observed in the epidermal cells co-bombarded with the YFP fusion proteins of LTSF2 and SKP1, indicating a weak interaction between LTSF2 and SKP1 (Figure 3C). Overall, these results indicate that both LTSF1 and LTSF2 interact with SKP1 in plant cells, and the detection of the BiFC signals in the nucleus, cytoplasm, and plasma membrane indicates that *LTSF1* and *LTSF2* may be involved in proteasome degradation in both the nucleus and the cytoplasm.

### 2.4. The Transgenic Expression of LTSF1 Promotes Low-Temperature Stress Tolerance in N. benthamiana Plants

**Figure 4:**
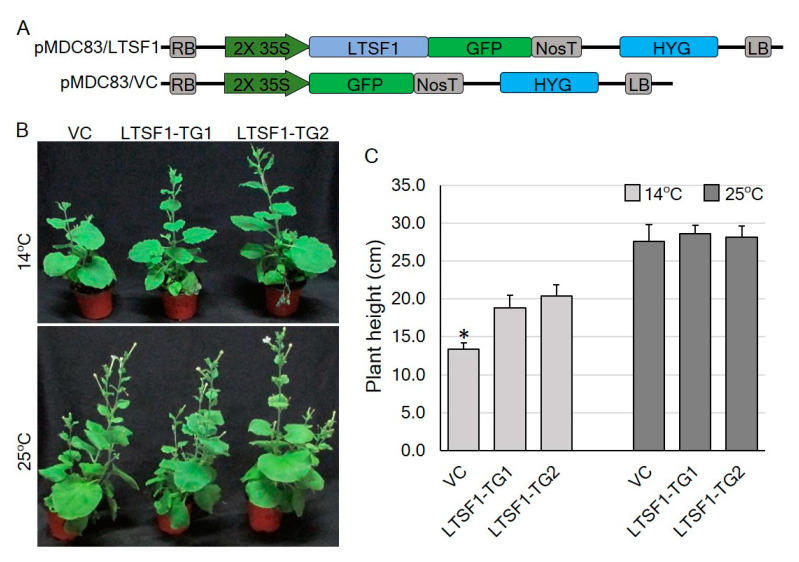
Transgenic *N. benthamiana* plants overexpressing *LTSF1*. (**A**) Schematic diagram of the transfer DNA (T-DNA) region of the pMDC83/VC and pMDC83/LTSF1 vectors: RB, right border of T-DNA region; LB, left border of T-DNA region; 2× 35S, Cauliflower Mosaic Virus 35S promoter; HYG, hygromycin phosphotransferase gene; NosT, nopaline synthase terminator. (**B**) Phenotype of the *LTSF1*-overexpressing and vector control (VC) transgenic *N. benthamiana* plants after 30 days of low-temperature stress (14 °C). (**C**) Plant heights of the *LTSF1-*overexpressing transgenic and VC plants under temperature stress (14 °C) and normal conditions (25 °C). The error bars indicate the mean ± SD of six biological replicates. * *p* < 0.01 vs. control (VC).

To confirm the role of the pepper F-box gene *LTSF1* in low-temperature stress tolerance, transgenic *N. benthamiana* lines overexpressing *LTSF1* were developed (Figure 4A,B). The genotypes of the T_0_ transgenic *LTSF1*-expressing lines were confirmed using reverse-transcription PCR (RT-PCR) analysis (Figure S2). Two transgenic lines (*LTSF1-TG1* and *-TG2*) and their corresponding vector control (VC) plants were grown under normal (25 °C) and low (14 °C) temperatures for one month. No significant morphological changes were observed between the *LTSF1*-overexpressing *N. benthamiana* lines and the control plants under the normal conditions; however, the reduced plant growth and shorter heights induced in the control plants by the low-temperature conditions were less severe in the transgenic lines, which grew approximately 1.5 times taller than the control plants (Figure 4B,C). Thus, the transgenic expression of *LTSF1* alleviated the effect of low-temperature stress in the *LTSF1-TG N. benthamiana* plants.

**Figure 5:**
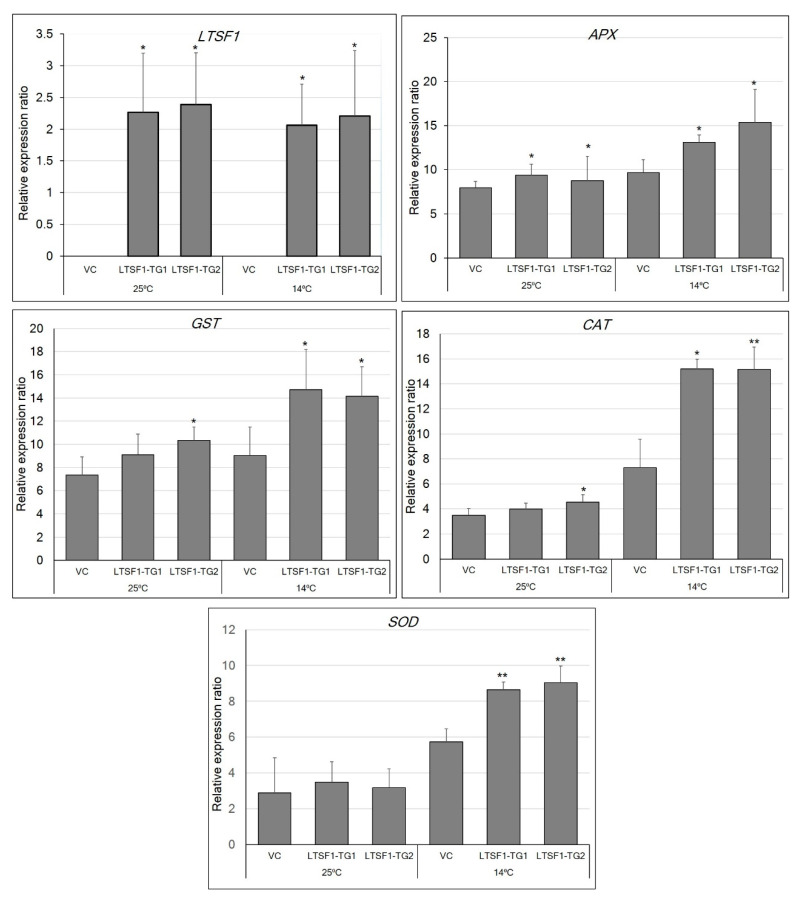
Relative expressions of levels of *LTSF1* and antioxidant-enzyme related genes (ascorbate peroxidase (APX), catalase (CAT), glutathione S-transferases (GST), and superoxide dismutase (SOD)) in transgenic *N. benthamiana* plants. The error bars indicate the mean ± SD of six biological replicates. * *p* < 0.05 and ** *p* < 0.01 vs. the vector control (VC).

To determine whether *LTSF1* regulates low-temperature stress through the antioxidant pathway, we analyzed the transcript abundances of the genes encoding ROS-scavenging antioxidant enzymes, such as ascorbate peroxidase (APX), catalase (CAT), glutathione S-transferase (GST), and superoxide dismutase (SOD), in the control and transgenic plants subjected to low-temperature stress. We observed a significant increase in the accumulation of *APX* transcripts in the transgenic *LTSF1-TG1* and *-TG2* plants compared with the control plants both under the stressed and non-stressed conditions (Figure 5). Similarly, the expression of *GST* and *CAT* was also significantly increased in the *LTSF1-TG1* and *-TG2* plants under the stress conditions. The accumulation of *SOD* transcripts was higher in the *LTSF1-TG1* and *-TG2* transgenic plants subjected to the stress conditions than in those grown in the non-stressed conditions. Overall, the expression levels of the genes encoding the ROS-scavenging enzymes were considerably altered by the low-temperature stress, and their levels were higher in the transgenic plants than in the control plants. These results indicate that the transgenic expression of *LTSF1* may influence the antioxidant system and, thus, confers low-temperature tolerance.

## 3. Discussion

The SCF complex is an E3 ubiquitin ligase that mediates the ubiquitination of proteins destined for proteasomal degradation in several plant developmental processes, including hormone signaling, morphogenesis, embryo development, circadian rhythms, and senescence [26]. F-box proteins are adaptor subunits of the SCF complex; the N-terminal F-box domain and the SKP subunit protein physically interact to form a core complex and recruit target proteins for proteasomal degradation through the C-terminal protein–protein interaction domain of the F-box protein [28,29,30].

In this study, the low-temperature stress-responsive *LTSF1* and *LTSF2* genes were predicted to encode F-box proteins containing a C-terminal FBA domain, which participates in protein–protein interactions associated with a wide range of plant physiological processes [31,32,33,34]. We cloned the F-box genes *LTSF1* and *LTSF2* from pepper and performed BiFC and Y2H assays to verify that LTSF1 and LTSF2 are functional F-box proteins. Protein–protein interaction studies revealed a weak interaction between LTSF2 and SKP1 compared with the LTSF1 and SKP1. The differences interaction between these proteins could be due to a single AA change (valine (V) in LTSF1 to alanine (A) in LTSF2) in their F-box domain (Figure 1).

To examine the subcellular localization of LTSF1 and LTSF2, we fused the LTSF1 and LTSF2 genes to the N-terminus of a green fluorescent protein (GFP) reporter gene and transformed the fusion protein expression vectors into *N. benthamiana* leaves for the transient expression of LTSF1/LTSF2:GFP fusion proteins. As shown in Figure S3, the green fluorescence signal was distributed throughout the cells infiltrated with the 35S::GFP control vector (Figure S3**)**. Similarly, the fluorescence signal of the 35S::LTSF1/2-GFP fusion was detected throughout the cells, including in the nucleus, cytoplasm, and plasma membrane (Figure S3). In previous studies, SCF complex proteins, such as the tomato E3 ubiquitin ligase SlBAH1, the *Arabidopsis* SKP1-like protein13 (AtASK13), the wheat (*Triticum aestivum*) F-box protein TaFBA1, and the rice F-box protein OsMAIF1, were found to be involved in the tolerance of biotic and abiotic stresses, and were also documented to be localized to the nucleus, cytoplasm, and plasma membrane [35,36,37,38]. The dispersed subcellular localizations of LTSF1, LTSF2, and other SCF complex proteins may, therefore, contribute to their diverse roles in plant cells.

Various components of SCF complexes have been reported to be associated with plant growth, development, and the responses to biotic and abiotic stresses [19,34,35,36,39,40] while a number of stress conditions have been documented to activate the expression of genes encoding the subunits of the SCF complex [28,41]. In the present study, to confirm the functional role of *LTSF1* and *LTSF2* genes in conveying low-temperature tolerance, we silenced the expression of these genes using VIGS. Silencing of the *LTSF1* or *LTSF2* gene by targeting their UTR region was not successful in our case. The double silencing of both *LTSF1* and *LTSF2* genes by targeting their CDS region resulted in an abnormal phenotype and severe growth retardation under low-temperature conditions (20 °C). Consistent with the suppressed expression of *LTSF1/LTSF2*, these double-silenced plants produced small, irregularly shaped, thick, and pale leaves (Figure 2), similar to those observed in the low-temperature-sensitive ‘*sy-2*’ cultivar grown under low temperatures [19]. Furthermore, VIGS of the LRR domain-containing *Capsicum annuum* *F-box* (*CaF-box*) gene reduced the cold-stress tolerance of pepper seedlings [42] supporting the role of the F-box genes in regulating the temperature sensitivity of plants.

To further confirm the role of *LTSF1* and *LTSF2* in low-temperature stress, we developed *LTSF1* overexpressing transgenic *N. benthamiana* lines. We observed that the growth of the vector control plants in terms of their height was more severely inhibited by low-temperature stress than in the transgenic lines overexpressing *LTSF1*. The transgenic plants showed enhanced transcriptional activity for the genes encoding the antioxidant enzymes, including SOD, APX, CAT, and GST (Figure 5), which could be associated with the improved temperature tolerance shown by the transgenic plants. Similar results were observed in transgenic wheat and tobacco plants overexpressing the *TaFBA1* F-box gene, in which increased antioxidant-enzyme activity was correlated with increased abiotic stress [37,43]. In another study, the overexpression of *SKP1* improved the resistance of *Arabidopsis* to various abiotic stresses, including heat, salinity, and oxidative stress [35], whereas the T-DNA insertion mutants and RNAi mutant lines lacking the related *ASK13* gene showed a low tolerance to abiotic stress [35]. Our previous study revealed that the cell death and leaf deformation observed in the ‘*sy*-*2*’ mutant grown under low temperatures is caused by the excessive accumulation of ROS, and is correlated with the downregulation of the genes encoding ROS-scavenging enzymes at 20 °C [19]. In the present study, *N. benthamiana* plants overexpressing *LTSF1* showed a markedly increased expression of ROS-scavenging enzyme-encoding genes, such *APX*, *CAT*, *GST*, and *SOD*, further substantiating the role of the ROS-scavenging pathway in low-temperature stress tolerance (Figure 5). The F-box protein LTSF1, which is a key subunit of the SCF complex, may, therefore, play an important role in positively regulating low-temperature tolerance by modulating the stress-related genes, possibly through the repression of the negative regulators of cold stress.

A previous study revealed that the cell death and leaf deformation observed in the ‘*sy*-*2*’ mutant grown under low temperatures is caused by the excessive accumulation of ROS, and is correlated with the downregulation of the genes encoding ROS-scavenging enzymes, such as *APX6*, *APXT1*, and *GST1*, at 20 °C [6]. Furthermore, salt-tolerant transgenic tobacco plants overexpressing the *TaFBA1* gene showed increased antioxidant-enzyme activity along with increased E3 ligase enzyme activity [44]. In the present study, *N. benthamiana* plants overexpressing *LTSF1* showed a markedly increased expression of ROS-scavenging enzyme-encoding genes, such *APX*, *CAT*, *GST*, and *SOD*, further substantiating the role of the ROS-scavenging pathway in low-temperature stress tolerance (Figure 5). However, further experiments will be needed to determine whether the increased transcriptional activity of antioxidant-enzyme genes in transgenic *N. benthamiana* plants overexpressing *LTSF1* could be associated with increased E3 ligase activity.

## 4. Materials and Methods

### 4.1. Plant Materials

For the functional characterization of the two F-box candidate genes using VIGS, the pepper (*C. chinense*) ‘No.3341’ (wild-type) and ‘*sy-2*’ (mutant) cultivars [19] were used. For the functional studies of the F-box genes using heterologous expression, tobacco (*N. benthamiana*) was used. Plants were maintained under a 16/8 h light–dark regime at 24 ± 2 °C.

### 4.2. Phylogenetic Analysis and Identification of Conserved Motifs

Homologs of *LTSF1* and *LTSF2* (Table S1) were identified using a Basic Local Alignment Search tool (BLAST) search of other plant genomes in the National Center for Biotechnology Information (NCBI) database with default parameters using the LTSF1 and LTSF2 protein sequences as queries. To verify the conserved domains, the F-box homologous protein sequences were compared with known sequences using the ClustalW2 alignment tool with default parameters [45]. A multiple-sequence alignment of the LTSF1 and LTSF2 proteins and their homologs from other plant species, including *Arabidopsis*, tomato (*Solanum lycopersicum*), and rice, was created using the ClustalW2 program with default parameters [28]. The InterPro protein sequence analysis and classification tool [46] was used for the identification of the functional domains.

### 4.3. VIGS

Total RNA was extracted from the pepper (‘No.3341’) leaf samples using an MG RNAzol kit (MGmed, Seoul, Korea), according to the manufacturer’s instructions. Complementary DNA (cDNA) was synthesized from 1 μg RNA using EasyScript Reverse Transcriptase kit (TransGen Biotech, Beijing, China). VIGS target sequences in the *LTSF1* and *LTSF2* genes were amplified from the cDNA sequences of pepper using Pfu DNA Taq polymerase (Bioneer, Seoul, Korea) and gene-specific primers (Table 1). The target sequence for silencing of *LTSF1* and *LTSF2* genes were predicted by sol genomics VIGS tool [47] via BLAST against the *Capsicum annuum* v1.55 genome. Ligation-independent cloning (LIC) was used to clone the VIGS target sequences (*LTSF1* (135 bp), *LTSF2* (127 bp), and *LTSF1/2* (305 bp)) into the *TRV* VIGS vector (TRV2), as previously described [48]. Plasmids from the positively transformed clones were isolated, purified, and sequenced at the National Instrumentation Center for Environmental Management (NICEM; Seoul National University, Seoul, Korea). The *Agrobacterium tumefaciens* strain *GV3101* was transformed with the pTRV2 (containing the target sequences) and pTRV1 vectors, then used for the coinfiltration of 3-week-old pepper cotyledons using a needleless syringe, as previously described [48]. Control plants infiltrated with empty vector (TRV2:00) and TRV1 were used as mock. Three weeks after coinfiltration of TRV constructs, quantitative measurements including leaf area and plant height were recorded. The VIGS experiment was repeated two times with least three biological replicates in each trail. ImageJ software was used for the analysis of leaf area.

### 4.4. Subcellular Localization of the LTSF1 and LTSF2 Proteins

The *LTSF1* coding sequence was amplified in a total volume of 50 μL using PrimeStar GXL DNA polymerase (Takara Bio, Kusatsu, Japan) with four-fold-diluted cDNA and 10 pmol of each gene-specific primer (Table 1). The PCR cycling conditions were as follows: initial denaturation at 95 °C for 3 min; followed by 34 cycles of denaturation at 98 °C for 10 s, annealing at 60 °C for 15 s, and extension at 68 °C for 1 min; with a final extension at 68 °C for 5 min. The *LTSF1* and *LTSF2* coding sequences, without the stop codon, were independently inserted into the pMDC83 expression vector at the *Spe*I-*Asc*I restriction sites using gene-specific primer pairs (Table 1), resulting in the fusion of green fluorescent protein (GFP) to the *C*-termini of *LTSF1* and *LTSF2*. The recombinant GFP constructs were agro-infiltrated into *N. benthamiana* leaves. Cells harboring an empty pMDC83-GFP vector were used as a control. At 48 h after agro-infiltration, the GFP signals were detected using a fluorescence microscope (Axioskop 2; Carl Zeiss, Oberkochen, Germany).

### 4.5. Y2H Assays

The coding sequences of *LTSF1*, *LTSF2*, and *SKP1* were cloned in frame into the *Eco*RI-*Bam*HI sites in the pGADT7 and pGBKT7 vectors to generate preys and baits, respectively, using the primers listed in Table 1. The paired constructs were co-transformed into the yeast strain *AH109* and grown on a -Leu/-Trp selection medium at 28 °C for 2–5 days. Positive yeast transformants were grown on SD/-Trp/-Leu/-His/3-AT selection medium for the interaction studies. The GUS assay was performed as described in the Yeast Protocols Handbook.

### 4.6. Bimolecular Fluorescence Complementation (BiFC) Analysis

BiFC analyses were conducted as described by Walter *et al*. [49]. For the BiFC vector constructs, *SKP1*, *LTSF1*, and *LTSF2* cDNAs lacking the termination codons were amplified using gene-specific PCR primers (listed in Table 1) and cloned into the *Eco*RI-*Bam*HI sites of the binary vectors pSPYNE and pSPYCE, which harbor sequences encoding the YFP^N^ and YFP^C^ (YFP N- and C-termini) protein fragments, respectively. The resulting constructs were named SKP1-YFP^N^, SKP1-YFP^C^, LTSF1-YFP^N^, LTSF1-YFP^C^, LTSF2-YFP^N^, and LTSF2-YFP^C^. The designated plasmid combinations were co-bombarded into onion (*Allium cepa*) epidermal cells using the Bio-Rad PDS 1000/He Biolistic Delivery System (Bio-Rad Laboratories, Hercules, CA, USA). On days 1 to 3 after transformation, the onion epidermal cells were visualized using an LSM5 Exciter confocal laser-scanning microscope (LSM 510; Carl Zeiss, Jena, Germany) at NICEM. YFP was excited using a 514 nm laser beam, and the emissions were detected at 525–600 nm. The BiFC experiment was repeated twice with six biological replicates in each experiment.

### 4.7. Genomic DNA Extraction

Genomic DNA was extracted from young *N. benthamiana* leaves using the cetyltrimethylammonium bromide method [48]. The concentration and quality of genomic DNA were analyzed using a Nanodrop spectrophotometer (BioTek, Winooski, VT, USA).

### 4.8. Agrobacterium-Mediated Transformation of N. benthamiana

The *pMDC83-LTSF1:GFP* vector constructed above was used for the *Agrobacterium*-mediated transformation of *N. benthamiana*, which was performed following the method described by Benvenuto et al. [50]. Putative transformants were confirmed using RT-PCR analyses with *LTSF1* and *Hpt* (*hygromycin phosphotransferase*) gene sequence-specific primers (Table 1). Total RNA was extracted from young *N. benthamiana* leaf samples and cDNA was synthesized as described below. RT-PCR was performed in a total volume of 25 μL containing 4 μL diluted cDNA template, 2.5 μL 10 × PCR buffer, 2.0 μL 2.5 mM dNTP mix, 0.5 μL each of 10 μM forward and reverse primers, 0.25 μL homemade Taq polymerase, and 15.25 μL of water. The PCR cycling conditions were as follows: initial denaturation at 94 °C for 5 min; followed by 32 cycles of denaturation at 94 °C for 60 s, annealing at 55 °C for 60 s, and extension at 72 °C for 60–90 s; with a final extension at 72 °C for 5 min. The identified T_0_ plants were micro-propagated using Murashige and Skoog [51] media to obtain multiple plants for the subsequent analysis.

### 4.9. Low-Temperature Stress Treatments

Approximately two-week-old transgenic and vector control (VC) *N. benthamiana* plants with a uniform appearance were grown in a walk-in growth chamber at 14 °C and 25 °C. Five plants per treatment were used in the stress experiments. After one month, the plant heights were recorded and leaf samples were collected for the qPCR analysis.

### 4.10. Total RNA Extraction and Reverse-Transcription Quantitative PCR (RT-qPCR)

To analyze the expression of *LTSF1* and *LTSF2*, systemic leaves were collected from the VIGS pepper plants. The confirmation of the expression of the transgenes and antioxidant enzyme-encoding genes in the *N. benthamiana* plants were also performed using leaf tissues. Total RNA was extracted from these samples using an MG RNAzol kit (MGmed, Seoul, Korea), according to the manufacturer’s instructions. The concentration and integrity of RNA were verified using 1% agarose gel electrophoresis and a Nanodrop spectrophotometer (BioTek, Winooski, VT, USA). A 1-μg aliquot of total RNA was used to produce cDNA using an EasyScript Reverse Transcriptase kit (TransGen Biotech, Beijing, China), according to the manufacturer’s instructions. The resulting cDNAs were used for further expression studies.

The qPCR analysis was performed using the primers listed in Table S2. The expression levels of genes encoding the antioxidant enzymes were analyzed using a Light Cycler 2.0 instrument (Roche, Basel, Switzerland) using the following conditions: 95 °C for 4 min; followed by 42 cycles of 95 °C for 10 s, 58 °C for 20 s, and 72 °C for 20 s. Three biological replicates and three technical replicates were used for each gene. The relative expression levels were calculated by normalizing the expression levels to the endogenous reference gene *Actin*. The experiment was repeated twice.

### 4.11. Statistical Analysis

The statistical significance of the data was tested using Student’s t-test, and the analyses were performed using Microsoft Excel 2016 (Microsoft, Redmond, WA, USA).

## 5. Conclusions

In conclusion, to elucidate the role of F-box genes in regulating the low-temperature sensitivity of pepper, we knocked down the expression of *LTSF1* and *LTSF2* genes using VIGS. The pepper plants lacking transcripts for both of these F-box genes exhibited an abnormal phenotype under low-temperature conditions, suggesting that they play an important function in plant adaptation to low-temperature stress. Furthermore, LTSF1 and LTSF2 function as part of the SCF complex, and could, therefore, modulate the degradation of the cellular regulatory proteins and enhance the plant response to low-temperature stress. Transgenic *N. benthamiana* plants overexpressing *LTSF1* showed less growth inhibition and the enhanced expression of genes encoding ROS-scavenging enzymes. The further functional analysis of the *LTSF1* and *LTSF2* genes and the identification of their novel protein–protein interaction partners and regulatory sites will be essential for the elucidation of their precise functional consequences.

## Figures and Tables

**Table 1:** Cloning primers used in this study.

Primer Name	Sequence	Purpose
Y2H: LTSF1/2: NdeF1	GAGACATATGATGCCTGTCAAAGTAGCA	Y2H vector cloning
Y2H: LTSF1/2: BamR1	GAGAGGATCCTTAGATAACTAATTTTGGAGA	Y2H vector cloning
Y2H: LTSF1/2: NdeF2	GAGACATATGATAGATTATGATCAGCAGGCAAT	Y2H vector cloning
Y2H: LTSF1/2: BamR2	GAGAGGATCCCTATACATCCTCAAAAGTGGATA	Y2H vector cloning
Y2H: LTSF1/2: NdeF3	TATACATATGGTACAACAACTCGATCCCCCT	Y2H vector cloning
SKP1-F-EcoRI	TCGCGAATTCATGTCTGCCCCAAAGAAAAT	Y2H vector cloning
SKP1-R-BamHI	TATAGGATCCTCACTCAAAGGCCCAAGCAT	Y2H vector cloning
SKP1:F-BamHI	CAATGGATCCATGTCTGCCCCAAAGAAAAT	BiFC vector cloning
SKP1:R-XhoI	ATATCTCGAGCTCAAAGGCCCAAGCA	BiFC vector cloning
LTSF1/2-F-BamHI	ACAGGGATCCATGCCTGTCAAAGTAGCA	BiFC vector cloning
LTSF1/2-R-XhoI	ACAGCTCGAGGATAACTAATTTTGGAGAA	BiFC vector cloning
VIGS-LTSF1UT-1F	CGACGACAAGACCCTAGGTGTTATTTTTGTCCTTTCC	TRV2 vector cloning
VIGS-LTSF1UT-1R	GAGGAGAAGAGCCCTCTGCAAACTCACTGTACAATTTAG	TRV2 vector cloning
VIGS-LTSF2UT-1F	CGACGACAAGACCCTGAAAAATGATTTTATGATATC	TRV2 vector cloning
VIGS-LTSF2UT-1R	GAGGAGAAGAGCCCTCAATGTATAATTTAGTCCACTGAAAAT	TRV2 vector cloning
VIGS-LTSF1/2-1F	CGACGACAAGACCCTTCCAAAACTTGGGAG	TRV2 vector cloning
VIGS-LTSF1/2-1R	GAGGAGAAGAGCCCTGCGGTTTCTCTTGTTAAGGGGT	TRV2 vector cloning
TRV2:LIC-F	TGTTACTCAAGGAAGCACGATGAGCT	LIC cloning confirmation
TRV2:LIC-R	CAGGCACGGATCTACTTAAAGAACGTAG	LIC cloning confirmation
LTSF1/2.Spe–F	GCTCACTAGTATGCCTGTCAAAGTAGCA	pMDC83 vector cloning/Transgene confirmation
LTSF1.Asc–R	AATATTGGCGCGCCAGATAACTAATTTTGGAGAA	
LTSF2.Asc–R	AATATTGGCGCGCCAAATAACTAATTTTGGAGAA	
Hpt-F	CCTGAACTCACCGCGACG	
Hpt-R	AAGACCAATGCGGAGCATAT	

LTSF1 and LTSF2—low-temperature-sensitive F-box proteins, Y2H—yeast two-hybrid, SKP1—S-phase kinase-associated protein 1, Hpt—hygromycin phosphotransferase, LIC—ligation-independent cloning, BiFC—bimolecular fluorescence complementation.

## Supplementary Materials

The following are available online at https://www.mdpi.com/2223-7747/9/9/1186/s1, Figure S1. Multiple sequence alignment of the LTSF1 and LTSF2 proteins from ‘No.3341’ and ‘sy-2’. Figure S2. RT-PCR analysis of N. benthamiana transgenic plants. TG-1 to TG5: LTSF1 N. benthamiana transgenic plants; Hpt, hygromycin phosphotransferase gene; M, molecular weight marker; WT, wild type N. benthamiana plant, Ve+, pMDC83-LTSF1:GFP vector. Figure S3. Subcellular localization of the LTSF1 and LTSF2 proteins. In N. benthamiana leaves transiently expressing 35S::LTSF1-GFP or 35S::LTSF2-GFP, the GFP signal was observed in the nucleus, cytoplasm, and cell membrane. pMDC83, empty GFP vector. Table S1. LTSF1 and LTSF2 cDNA sequences. Table S2. qPCR primers used in this study. Reference [52] is cited in supplementary Table S2.

## References

[1] Chinnusamy V., Zhu J.K., Sunkar R. (2010). Gene regulation during cold stress acclimation in plants. Methods Mol. Biol..

[2] Venkatesh J., Kang B.C. (2019). Current views on temperature-modulated R gene-mediated plant defense responses and tradeoffs between plant growth and immunity. Curr. Opin. Plant Biol..

[3] Korres N.E., Norsworthy J.K., Tehranchian P., Gitsopoulos T.K., Loka D.A., Oosterhuis D.M., Gealy D.R., Moss S.R., Burgos N.R., Miller M.R. (2016). Cultivars to face climate change effects on crops and weeds: A review. Agr. Sust. Devel..

[4] Thomashow M.F. (1999). Plant cold acclimation: Freezing tolerance genes and regulatory mechanisms. Annu. Rev. Plant Physiol..

[5] Van Heerden P.D., Strasser R.J., Kruger G.H. (2004). Reduction of dark chilling stress in N-fixing soybean by nitrate as indicated by chlorophyll a fluorescence kinetics. Physiol. Plant..

[6] An S.J., Pandeya D., Park S.W., Li J.J., Kwon J.K., Koeda S., Hosokawa M., Paek N.C., Choi D., Kang B.C. (2011). Characterization and genetic analysis of a low-temperature-sensitive mutant, *sy-2*, in *Capsicum chinense*. Theor. Appl. Genet..

[7] Koeda S., Hosokawa M., Kang B.C., Tanaka C., Choi D., Sano S., Shiina T., Doi M., Yazawa S. (2012). Defense response of a pepper cultivar cv. Sy-2 is induced at temperatures below 24 °C. J. Plant Res..

[8] Koeda S., Hosokawa M., Saito H., Doi M. (2013). Temperature-sensitive phenotype caused by natural mutation in *Capsicum* latescent in two tropical regions. J. Plant Res..

[9] Almadanim M.C., Alexandre B.M., Rosa M.T., Sapeta H., Leitão A.E., Ramalho J.C., Lam T.T., Negrão S., Abreu I.A., Oliveira M.M. (2017). Rice calcium-dependent protein kinase OsCPK17 targets plasma membrane intrinsic protein and sucrose-phosphate synthase and is required for a proper cold stress response. Plant Cell Environ..

[10] Domon J.M., Baldwin L., Acket S., Caudeville E., Arnoult S., Zub H., Gillet F., Lejeune-Hénaut I., Brancourt-Hulmel M., Pelloux J. (2013). Cell wall compositional modifications of Miscanthus ecotypes in response to cold acclimation. Phytochemistry.

[11] Kielbowicz-Matuk A., Rey P., Rorat T. (2008). The organ-dependent abundance of a Solanum lipid transfer protein is up-regulated upon osmotic constraints and associated with cold acclimation ability. J. Exp. Bot..

[12] Orvar B.L., Sangwan V., Omann F., Dhindsa R.S. (2000). Early steps in cold sensing by plant cells: The role of actin cytoskeleton and membrane fluidity. Plant J..

[13] Miquel M.F., Browse J.A. (1994). High-oleate oilseeds fail to develop at low temperature. Plant Physiol..

[14] Zhang J., Liu H., Sun J., Li B., Zhu Q., Chen S., Zhang H. (2012). *Arabidopsis* Fatty Acid Desaturase FAD2 is required for salt tolerance during seed germination and early seedling growth. PLoS ONE.

[15] Havaux M., Kloppstech K. (2001). The protective functions of carotenoid and flavonoid pigments against excess visible radiation at chilling temperature investigated in *Arabidopsis*
*npq* and *tt* mutants. Planta.

[16] Hua J., Grisafi P., Cheng S.H., Fink G.R. (2001). Plant growth homeostasis is controlled by the *Arabidopsis*
*BON1* and *BAP1* genes. Genes Dev..

[17] Millerd A., McWilliam J.R. (1968). Studies on a maize mutant sensitive to low temperature I. Influence of temperature and light on the production of chloroplast pigments. Plant Physiol..

[18] Peng Y., Zhang Y., Lv J., Zhang J., Li P., Shi X., Wang Y., Zhang H., He Z., Teng S. (2012). Characterization and fine mapping of a novel rice albino mutant *low temperature albino 1*. J. Genet. Genom..

[19] Liu L., Venkatesh J., Jo Y.D., Koeda S., Hosokawa M., Kang J.H., Goritschnig S., Kang B.C. (2016). Fine mapping and identification of candidate genes for the *sy-2* locus in a temperature-sensitive chili pepper (*Capsicum chinense*). Theor. Appl. Genet..

[20] Smalle J., Vierstra R.D. (2004). The ubiquitin 26S proteasome proteolytic pathway. Annu. Rev. Plant Biol..

[21] Lechner E., Achard P., Vansiri A., Potuschak T., Genschik P. (2006). F-box proteins everywhere. Curr. Opin. Plant Biol..

[22] Xu G., Ma H., Nei M., Kong H. (2009). Evolution of F-box genes in plants: Different modes of sequence divergence and their relationships. Proc. Natl. Acad. Sci. USA.

[23] Yang X., Kalluri U.C., Jawdy S., Gunter L.E., Yin T., Tschaplinski T.J., Weston D.J., Ranjan P., Tuskan G.A. (2008). The F-box gene family is expanded in herbaceous annual plants relative to woody perennial plants. Plant Physiol..

[24] Gagne J.M., Downes B.P., Shiu S.H., Durski A.M., Vierstra R.D. (2002). The F-box subunit of the SCF E3 complex is encoded by a diverse super family of genes in Arabidopsis. Proc. Natl. Acad. Sci. USA.

[25] Jain M., Nijhawan A., Arora R., Agarwal P., Ray S., Sharma P., Kapoor S., Tyagi A.K., Khurana J.P. (2007). F-box proteins in rice. Genome-wide analysis, classification, temporal and spatial gene expression during panicle and seed development, and regulation by light and abiotic stress. Plant Physiol..

[26] Stefanowicz K., Lannoo N., Van Damme E.J. (2015). Plant F-box proteins-judges between life and death. Crit. Rev. Plant Sci..

[27] Chae E., Tan Q.K., Hill T.A., Irish V.F. (2008). An Arabidopsis F-box protein acts as a transcriptional co-factor to regulate floral development. Development.

[28] Chen Y., Xu Y.Y., Luo W., Li W.X., Chen N., Zhang D.J., Chong K. (2013). The F-Box protein OsFBK12 targets OsSAMS1 for degradation and affects pleiotropic phenotypes, including leaf senescence, in rice. Plant Physiol..

[29] Potuschak T., Lechner E., Parmentier Y., Yanagisawa S., Grava S., Koncz C., Genschik P. (2003). EIN3-dependent regulation of plant ethylene hormone signaling by two Arabidopsis F box proteins: EBF1 and EBF2. Cell.

[30] Sonnberg S., Fleming S.B., Mercer A.A. (2009). A truncated two-alpha-helix F-box present in poxvirus ankyrin-repeat proteins is sufficient for binding the SCF1 ubiquitin ligase complex. J. Gen. Virol..

[31] Song J.B., Wang Y.X., Li H.B., Li B.W., Zhou Z.S., Gao S., Yang Z.M. (2015). The F-box family genes as key elements in response to salt, heavy mental, and drought stresses in *Medicago truncatula*. Funct. Integr. Genomic..

[32] Takahara M., Magori S., Soyano T., Okamoto S., Yoshida C., Yano K., Sato S., Tabata S., Yamaguchi K., Shigenobu S. (2013). Too much love, a novel Kelch repeat-containing F-box protein, functions in the long-distance regulation of the legume-Rhizobium symbiosis. Plant Cell Physiol..

[33] Takase T., Nishiyama Y., Tanihigashi H., Ogura Y., Miyazaki Y., Yamada Y., Kiyosue T. (2011). LOV KELCH PROTEIN2 and ZEITLUPE repress Arabidopsis photoperiodic flowering under non-inductive conditions, dependent on FLAVIN-BINDING KELCH REPEAT F-BOX1. Plant J..

[34] Zhang X., Gou M., Liu C.J. (2013). *Arabidopsis* Kelch repeat F-box proteins regulate phenylpropanoid biosynthesis via controlling the turnover of phenylalanine ammonialyase. Plant Cell.

[35] Rao V., Petla B.P., Verma P., Salvi P., Kamble N.U., Ghosh S., Kaur H., Saxena S.C., Majee M. (2018). Arabidopsis SKP1-like protein13 (ASK13) positively regulates seed germination and seedling growth under abiotic stress. J. Exp. Bot..

[36] Yan Y.S., Chen X.Y., Yang K., Sun Z.X., Fu Y.P., Zhang Y.M., Fang R.X. (2011). Overexpression of an F-box protein gene reduces abiotic stress tolerance and promotes root growth in rice. Mol. Plant.

[37] Zhou S.M., Kong X.Z., Kang H.H., Sun X.D., Wang W. (2015). The involvement of wheat F-box protein gene TaFBA1 in the oxidative stress tolerance of plants. PLoS ONE.

[38] Zhou S.M., Wang S.H., Lin C., Song Y.Z., Zheng X.X., Song F.M., Zhu C.X. (2016). Molecular cloning and functional characterisation of the tomato E3 ubiquitin ligase *SlBAH1* gene. Funct. Plant Biol..

[39] Bu Q., Lv T., Shen H., Luong P., Wang J., Wang Z., Huang Z., Xiao L., Engineer C., Kim T.H. (2014). Regulation of drought tolerance by the F-box protein MAX2 in *Arabidopsis*. Plant Physiol..

[40] Gray W.M., del Pozo J.C., Walker L., Hobbie L., Risseeuw E., Banks T., Crosby W.L., Yang M., Ma H., Estelle M. (1999). Identification of an SCF ubiquitin–ligase complex required for auxin response in *Arabidopsis thaliana*. Genes Dev..

[41] Liu Y.C., Wu Y.R., Huang X.H., Sun J., Xie Q. (2011). AtPUB19, a U-Box E3 ubiquitin ligase, negatively regulates abscisic acid and drought responses in *Arabidopsis thaliana*. Mol. Plant.

[42] Chen R.G., Guo W.L., Yin Y.X., Gong Z.H. (2014). Novel F-box protein CaF-box is involved in responses to plant hormones and abiotic stress in pepper (*Capsicum annuum* L.). Int. J. Mol. Sci..

[43] Zhou S., Sun X., Yin S., Kong X., Zhou S., Xu Y., Luo Y., Wang W. (2014). The role of the F-box gene *TaFBA1* from wheat (*Triticum aestivum* L.) in drought tolerance. Plant Physiol. Biochem..

[44] Zhao Z., Zhang G., Zhou S., Ren Y., Wang W. (2017). The improvement of salt tolerance in transgenic tobacco by overexpression of wheat F-box gene *TaFBA1*. Plant Sci..

[45] Larkin M.A., Blackshields G., Brown N.P., Chenna R., McGettigan P.A., McWilliam H., Valentin F., Wallace I.M., Wilm A., Lopez R. (2007). Clustal W and Clustal X version 2.0. Bioinformatics.

[46] Mitchell A., Chang H.Y., Daugherty L., Fraser M., Hunter S., Lopez R., McAnulla C., McMenamin C., Nuka G., Pesseat S. (2015). The InterPro protein families database: The classification resource after 15 years. Nucleic Acids Res..

[47] Fernandez-Pozo N., Rosli H.G., Martin G.B., Mueller L.A. (2015). The SGN VIGS Tool: User-friendly software to design virus-induced gene silencing (VIGS) constructs for functional genomics. Mol. Plant.

[48] Hwang J., Lee S., Lee J.H., Kang W.H., Kang J.H., Kang M.Y., Oh C.S., Kang B.C. (2015). Plant translation elongation factor 1Bβ facilitates potato virus x (PVX) infection and interacts with PVX triple gene block protein 1. PLoS ONE.

[49] Walter M., Chaban C., Schütze K., Batistic O., Weckermann K., Näke C., Blazevic D., Grefen C., Schumacher K., Oecking C. (2004). Visualization of protein interactions in living plant cells using bimolecular fluorescence complementation. Plant J..

[50] Benvenuto E., Ordàs R.J., Tavazza R., Ancora G., Biocca S., Cattaneo A., Galeffi P. (1991). ‘Phytoantibodies’: A general vector for the expression of immunoglobulin domains in transgenic plants. Plant Mol. Biol..

[51] Murashige T., Skoog F. (1962). A revised medium for rapid growth and bio assays with tobacco tissue cultures. Physiol. Plant..

[52] Li Y., Zhang L., Wang X., Zhang W., Hao L., Chu X., Guo X. (2013). Cotton GhMPK6a negatively regulates osmotic tolerance and bacterial infection in transgenic Nicotiana benthamiana, and plays a pivotal role in development. FEBS J..

