# Supplementary material for: F-Box Family Genes, LTSF1 and LTSF2, Regulate Low-Temperature Stress Tolerance in Pepper (Capsicum chinense)
Source: Plants (Basel). 2020 Sep 11;9(9):1186. doi: 10.3390/plants9091186 (PMC7570372; doi:10.3390/plants9091186)
Supplement: Supplementary file 1 [file plants-09-01186-s001.zip › Supplementary Figures.pdf]

```
No.3341-LTSF2       MPVKVAKRKHFQTENGFSQSEQQMEKQRIPPIIQIQPYNEAKKVDLESRKKANILKCQQQ 60
sy2-LTSF2           MPVKVAKRKHFQTENGFSQSEQQMEKQRIPPIIQIQPYNEAKKVDLESRKKANILKCQQQ 60
No.3341-LTSF1       MPVKVAKRKHFQTENGFSQSEQQMEKQRIPPIIQIQPYNEAKKVDLESRKKANILKCQHQ 60
sy-2-LTSF1          MPVKVAKRKHFQTENGFSQSEQQMEKQRIPPIIQIQPYNEAKKVDLESRKKANILKCQHQ 60
                    **********************************************************:*

No.3341-LTSF2       GKKKLLKKKSVPHGQIRKGKRVVKKIDYDQQAIHFQEQILVDILSRLPLRSLLRFKCASK 120
sy2-LTSF2           GKKKLLKKKSVPHGQIRKGKRVVKKIDYDQQAIHFQEQILVDILSRLPLRSLLRFKCASK 120
No.3341-LTSF1       GKKKLLKKKSVPHGQIRKGKRVVKKIDYDQQAIHFQEQILVDILSRLPLRSLLRFKCVSK 120
sy-2-LTSF1          GKKKLLKKKSVPHGQIRKGKRVVKKIDYDQQAIHFQEQILVDILSRLPLRSLLRFKCVSK 120
                    *********************************************************.**

No.3341-LTSF2       TWEALISDPYFHKMIMKKQRQSPDSTSQKLLIRKMFSDTAPSRFKFYSSSLSTFEDVQQL 180
sy2-LTSF2           TWEALISDPYFHKMIMKKQRQSPDSTSQKLLIRKMFSDTAPSRFKFYSSSLSTFEDVQQL 180
No.3341-LTSF1       TWEALISDPYFHKMIMKKQRQSPDSTSQKLLIRKMFSDTAPSRFKFYSSSLSTFEDVQQL 180
sy-2-LTSF1          TWEALISDPYFHKMIMKKQRQSPDSTSQKLLIRKMFSDTAPSRFKFYSSSLSTFEDVQQL 180
                    ************************************************************

No.3341-LTSF2       DPPLTHYVDRYHIFSSFDGLALLAADTQLFLWNPLTRETALLPHTEFEAMCTCFVLGYDD 240
sy2-LTSF2           DPPLTHYVDRYHIFSSFDGLALLAADTQLFLWNPLTRETALLPHTEFEAMCTCFVLGYDD 240
No.3341-LTSF1       DPPLTHYVDRYHIFSSFDGLALLSADTQLFLWNPLTRETALLPHTEFEAMCTCFVLGYDD 240
sy-2-LTSF1          DPPLTHYVDRYHIFSSFDGLALLSADTQLFLWNPLTRETALLPHTEFEAMGTCFVLGYDD 240
                    ***********************:************************** *********

No.3341-LTSF2       ATDGYKILKFVMRLQSSMEILTLKTGLWRKICNYPTAVCPTTGHEMEIIPRPSKNIPMEP 300
sy2-LTSF2           ATDGYKILKFVMRLQKSMEILTLKTGLWRKICNYPTAVCPTTGHEMEIIPRPSKNIPMEP 300
No.3341-LTSF1       ATDGYKILKFVMGLQKSMEILTLKTGFWRKICNHPTAVCPTTGHEMEILPRPSKNIPMEP 300
sy-2-LTSF1          ATDGYKILKFVMGLQKSMEILTLKTGFWRKICNHPTAVCPTTGHEMEILPRPSKNIPMEP 300
                    ************ **.**********:******:**************:***********

No.3341-LTSF2       LVCVHGAFHWLGDLKVSGKFYVVSFSISNEVYGDVPLLEEMGKKVNYRPDFGLSLLDGML 360
sy2-LTSF2           LVCVHGAFHWLGDLKVSGKFYVVSFSISNEVYGDVPLLEEMGKKVNYRPDFGLSLLDGML 360
No.3341-LTSF1       LVCVHGAFHWLGDLNVSGKFYVVSFSISNEVYGDVPLLEEMGKKVNYRPDFGLSLLDGML 360
sy-2-LTSF1          LVCVHGAFHWLGDLDVSGKFYVVSFSISNEVYGDVPLLEEMGKKVNYRPDFGLSLLDGML 360
                    **************.*********************************************

No.3341-LTSF2       CYYTTTNDGSLGSFKLWAMKDYGVKESWIVLCTLQFNGLVIAKPRYRFPDGGLLLYAPGE 420
sy2-LTSF2           CYYTTTNDGSLGSFKLWAMKDYGVKESWIVLCTLQFNGLVIAKPRYRFPDGGLLLYAPGE 420
No.3341-LTSF1       CYYTTTNDGSLGSFKLWAMKDYGVKESWIVLCTLQFNGLVIAKPRYRFPDGTFAPAIKCR 420
sy-2-LTSF1          CYYTTTNDGSLGSFKLWAMKDYGVKESWIVLCTLQFNGLVIAKPRYRFPDGTFAPAIKCR 420
                    *************************************************** :      .

No.3341-LTSF2       FPHQCGVPSCFTF-------QTFKGKFGFWPERGGTFEKGIVYRESLISPKLVI       467
sy2-LTSF2           FPHQCGVPSCFTF-------QTFKGKFGFWPERGGTFEKGIVYRESLISPKLVI       467
No.3341-LTSF1       PLVLMSVACKFSFTEVACVFYALASYLWLW------------------------       450
sy-2-LTSF1          PLVLMSVACKFSFTEVACVFYALASYLWLW------------------------       450
                         .* . *:*        :: . : :*
```

Figure S1. Multiple sequence alignment of the LTSF1 and LTSF2 proteins from 'No.3341' and '*sy-2*'.

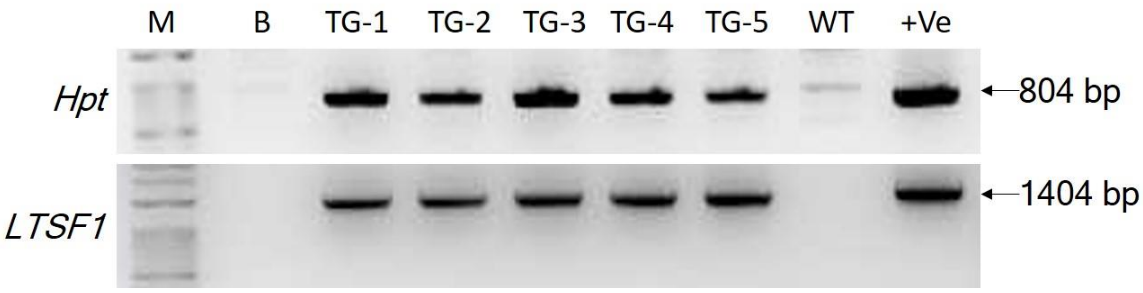

Figure S2. RT-PCR analysis of *N. benthamiana* transgenic plants. TG-1 to TG5: *LTSF1 N. benthamiana* transgenic plants; *Hpt*, hygromycin phosphotransferase gene; M, molecular weight marker; WT, wild type *N. benthamiana* plant, Ve+, pMDC83-*LTSF1:GFP* vector.

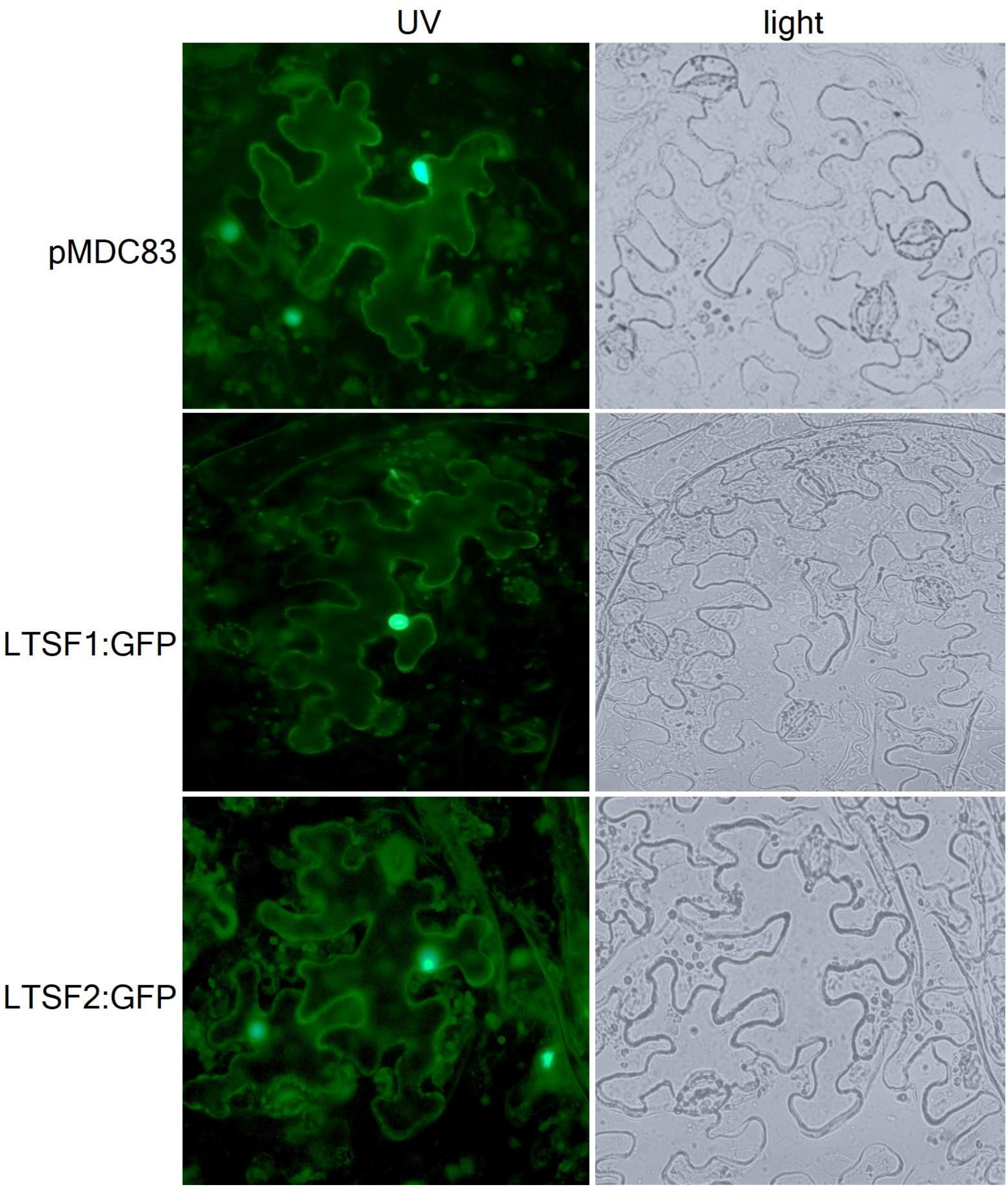

Figure S3. Subcellular localization of the LTSF1 and LTSF2 proteins. In *N. benthamiana* leaves transiently expressing *35S::LTSF1-GFP* or *35S::LTSF2-GFP*, the GFP signal was observed in the nucleus, cytoplasm, and cell membrane. pMDC83, empty GFP vector.
