# Supplementary material for: F-Box Family Genes, LTSF1 and LTSF2, Regulate Low-Temperature Stress Tolerance in Pepper (Capsicum chinense)
Source: Plants (Basel). 2020 Sep 11;9(9):1186. doi: 10.3390/plants9091186 (PMC7570372; doi:10.3390/plants9091186)
Supplement: Supplementary file 1 [file plants-09-01186-s001.zip › Table S1.pdf]

Table S1. *LTSF1* and *LTSF2* cDNA sequences.

>No.3341-LTSF1<br>ATGCCTGTCAAAGTAGCAAAGAGAAAGCATTTCCAAACTGAAAATGGATTTTCTCAGTCAGAGCAGCAAATGGAGAAGCAGAGAATCCCCCCTAT<br>CATTCAGATCCAACCATATAACGAAGCAAAAAAGGTAGATCTTGAATCGAGAAAAAAGGCAAATATTTTGAAATGCCAACATCAGGGGAAGAAG<br>AAGCTGTTGAAGAAGAAATCAGTACCTCACGGTCAAATTCGCAAGGGGAAGAGAGTCGTGAAGAAGATAGATTATGATCAGCAGGCAATTCATTT<br>CCAAGAGCAAATACTTGTGGATATCCTCAGCAGGTTACCCTTGCGCTCTCTTCTTCGCTTCAAATGTGTCTCCAAAACTTGGGAGGCATTAATATCA<br>GATCCTTATTTTCATAAGATGATAATGAAGAAGCAGCGTCAGAGTCCTGATTCAACTTCCCAAAAGCTTCTTATTCGCAAAATGTTCAGTGACACTG<br>CTCCCTCAAGGTTTAAATTCTATTCTTCTTCTTTATCCACTTTTGAGGATGTACAACAACTCGATCCCCCTTTAACCCACTATGTGGATCGTTATCACA<br>TCTTTTCTTCTTTCGACGGCTTGGCTCTTCTTTCTGCTGATACACAGCTTTTCTTGTGGAACCCCTTAACAAGAGAAACCGCACTACTTCCCCATACAG<br>AATTTGAGGCAATGTGCACTTGTTTCGTATTGGGATATGATGATGCCACTGATGGCTATAAGATCCTTAAATTGGACATGCGTCTACAAAAATCCAT<br>GGAAATTCTCACGCTTAAAACAAGGCTTTGGAGGAAAATTTGTAACCATCCAACTGCCGTTTGCCCTACGACAGGACATGAAATGGAGATTCTTCC<br>TCGCCCAAGTAAAAATATTCCTATGGAGCCTTTGGTATGTGTACATGGAGCTTTTCATTGGCTTGGTGATTTGAACGTATTTGGAAAGTTTTATGTGG<br>TTTCATTTAGTATTTCAAATGAGGTGTACGGAGACGTACCTTTGCTAGAGGAGATGGGGAAAAAGGTTAACTACCGCCCAGATTTTGGCCTTTCATT<br>ATTGGATGGAATGCTTTGCTATTATACTACTACTAATGATGGTTCATTGGGTTCTTTTAAGTTGTGGGCTATGAAAGACTATGGTGTCAAGGAATCTT<br>GGATAGTATTGTGTACCTTACAATTCAATGGTCTTGTTATAGCCAAACCGCGATATAGGTTTCCAGATGGTGAGCTGCTACTATTTTCACCGGGGGA<br>ATTCCCACATCAATGTGGGGTAGTTAGCTGTTTTACATTCCAGACATCCAAAGGAAAATTTGGATTTTGGCCTGAACGCGGAGGACCCTTTGAGAA<br>AGGAATCATTTATAGAGAAAGCTTGATTTCTCCAAAATTAGTTATCTAA
>sy2-LTSF1<br>ATGCCTGTCAAAGTAGCAAAGAGAAAGCATTTCCAAACTGAAAATGGATTTTCTCAGTCAGAGCAGCAAATGGAGAAGCAGAGAATCCCCCCTAT<br>CATTCAGATCCAACCATATAACGAAGCAAAAAAGGTAGATCTTGAATCGAGAAAAAAGGCAAATATTTTGAAATGCCAACATCAGGGGAAGAAG<br>AAGCTGTTGAAGAAGAAATCAGTACCTCACGGTCAAATTCGCAAGGGGAAGAGAGTCGTGAAGAAGATAGATTATGATCAGCAGGCAATTCATTT<br>CCAAGAGCAAATACTTGTGGATATCCTCAGCAGGTTACCCTTGCGCTCTCTTCTTCGCTTCAAATGTGTCTCCAAAACTTGGGAGGCATTAATATCA<br>GATCCTTATTTTCATAAGATGATAATGAAGAAGCAGCGTCAGAGTCCTGATTCAACTTCCCAAAAGCTTCTTATTCGCAAAATGTTCAGTGACACTG<br>CTCCCTCAAGGTTTAAATTCTATTCTTCTTCTTTATCCACTTTTGAGGATGTACAACAACTCGATCCCCCTTTAACCCACTATGTGGATCGTTATCACA<br>TCTTTTCTTCTTTCGACGGCTTGGCTCTTCTTTCTGCTGATACACAGCTTTTCTTGTGGAACCCCTTAACAAGAGAAACCGCACTACTTCCCCATACAG<br>AATTTGAGGCAATGGGCACTTGTTTCGTATTGGGATATGATGATGCCACTGATGGCTATAAGATCCTTAAATTGGACATGCGTCTACAAAAATCCAT<br>GGAAATTCTCACGCTTAAAACAAGGCTTTGGAGGAAAATTTGTAACCATCCAACTGCCGTTTGCCCTACGACAGGACATGAAATGGAGATTCTTCC<br>TCGCCCAAGTAAAAATATTCCTATGGAGCCTTTGGTATGTGTACATGGAGCTTTTCATTGGCTTGGTGATTTGGACGTATTTGGAAAGTTTTATGTGG<br>TTTCATTTAGTATTTCAAATGAGGTGTACGGAGACGTACCTTTGCTAGAGGAGATGGGGAAAAAGGTTAACTACCGCCCAGATTTTGGCCTTTCATT<br>ATTGGATGGAATGCTTTGCTATTATACTACTACTAATGATGGTTCATTGGGTTCTTTTAAGTTGTGGGCTATGAAAGACTATGGTGTCAAGGAATCTT<br>GGATAGTATTGTGTACCTTACAATTCAATGGTCTTGTTATAGCCAAACCGCGATATAGGTTTCCAGATGGTGAGCTGCTACTATTTTCACCGGGGGA<br>ATTCCCACATCAATGTGGGGTAGTTAGCTGTTTTACATTCCAGACATCCAAAGGAAAATTTGGATTTTGGCCTGAACGCGGAGGACCCTTTGAGAA<br>AGGAATCATTTATAGAGAAAGCTTGATTTCTCCAAAATTAGTTATCTAA
>No.3341-LTSF2<br>ATGCCTGTCAAAGTAGCAAAGAGAAAGCATTTCCAAACTGAAAATGGATTTTCTCAGTCAGAGCAGCAAATGGAGAAGCAGAGAATCCCCCCTAT<br>CATTCAGATCCAACCATATAACGAAGCAAAAAAGGTAGATCTTGAATCGAGAAAAAAGGCAAATATTTTGAAATGCCAACACCAGGGGAAGAAG<br>AAGCTGTTGAAGAAGAAATCAGTACCTCACGGTCAAATTCGCAAGGGGAAGAGAGTCGTGAAGAAGATAGATTATGATCAGCAGGCAATTCATTT

CCAAGAGCAAATACTTGTGGATATCCTCAGCAGGTTACCCTTGCGCTCTCTTCTTCGCTTCAAATGTGCCTCCAAAACTTGGGAGGCATTAATATCA
GATCCTTATTTTCATAAGATGATAATGAAGAAGCAGCGTCAGAGTCCTGATTCAACTTCCCAAAAGCTTCTTATTCGCAAAATGTTCAGTGACACTG
CTCCCTCAAGGTTTAAATTCTATTCTTCTTCTTTATCCACTTTTGAGGATGTACAACAACTCGATCCCCCTTTAACCCACTATGTGGATCGTTATCACA
TCTTTTCTTCTTTCGACGGCTTGGCTCTTCTTGCTGCTGATACACAGCTTTTCTTGTGGAACCCCTTGACAAGAGAAACCGCACTACTTCCCCATACAG
AATTTGAGGCAATGTGCACTTGTTTCGTATTGGGATATGATGATGCCACTGATGGCTATAAGATCCTTAAATTTGTCATGCGTCTACAAAGTTCCATG
GAAATTCTCACGCTTAAAACAGGGCTTTGGAGGAAAATTTGTAACTATCCAACTGCCGTTTGCCCTACGACAGGACATGAAATGGAAATTATTCCT
CGCCCAAGTAAAAATATTCCTAGGGAGCCTTTGGTATGTGTACATGGAGCTTTTCATTGGCTTGGTGATTTGAAGGTATCAGGAAAGTTTTATGTGG
TTTCATTTAGTATTTCAAATGAGGTGTACGGAGACGTACCTTTGCTAGAGGAGATGGGGAAAAAGGTTAACTACCGCCCAGATTTTGGCCTTTCATT
ATTGGATGGAATGCTTTGCTATTATACTACTACTAATGATGGTTCATTGGGTTCTTTTAAGTTGTGGGCTATGAAAGACTATGGTGTCAAGGAATCTT
GGATAGTATTGTGTACCTTACAATTCAATGGTCTTGTTATAGCCAAACCACGATATAGGTTTCCAGATGGTGAGCTGCTACTATTTTCACCGGGGGA
ATTCCCACATCAATGTGGGGTAGTTAGCTGTTTTACATTCCAGACATCCAAAGGAAAATTTGGATTTTGGCCTGAACGCGGAGGAACCTTTGAGAA
AGGAATCATTTATAGAGAAAGCTTGATTTCTCCAAAATTAGTTATCTAG

>sy2-LTSF2
ATGCCTGTCAAAGTAGCAAAGAGAAAGCATTTCCAAACTGAAAATGGATTTTCTCAGTCAGAGCAGCAAATGGAGAAGCAGAGAATCCCCCCTAT
CATTCAGATCCAACCATATAACGAAGCAAAAAAGGTAGATCTTGAATCGAGAAAAAAGGCAAATATTTTGAAATGCCAACAGCAGGGGAAGAAG
AAGCTGTTGAAGAAGAAATCAGTACCTCACGGTCAAATTCGCAAGGGGAAGAGAGTCGTGAAGAAGATAGATTATGATCAGCAGGCAATTCATTT
CCAAGAGCAAATACTTGTGGATATCCTCAGCAGGTTACCCTTGCGCTCTCTTCTTCGCTTCAAATGTGCCTCCAAAACTTGGGAGGCATTAATATCA
GATCCTTATTTTCATAAGATGATAATGAAGAAGCAGCGTCAGAGTCCTGATTCAACTTCCCAAAAGCTTCTTATTCGCAAAATGTTCAGTGACACTG
CTCCCTCAAGGTTTAAATTCTATTCTTCTTCTTTATCCACTTTTGAGGATGTACAACAACTCGATCCCCCTTTAACCCACTATGTGGATCGTTATCACA
TCTTTTCTTCTTTCGACGGCTTGGCTCTTCTTGCTGCTGATACACAGCTTTTCTTGTGGAACCCCTTGACAAGAGAAACCGCACTACTTCCCCATACAG
AATTTGAGGCAATGTGCACTTGTTTCGTATTGGGATATGATGATGCCACTGATGGCTATAAGATCCTTAAATTTGTCATGCGTCTACAAAAATCCAT
GGAAATTCTCACGCTTAAAACAGGGCTTTGGAGGAAAATTTGTAACTATCCAACTGCCGTTTGCCCTACGACAGGACATGAAATGGAAATTATTCC
TCGCCCAAGTAAAAATATTCCTAGGGAGCCTTTGGTATGTGTACATGGAGCTTTTCATTGGCTTGGTGATTTGAAGGTATCAGGAAAGTTTTATGTG
GTTTCATTTAGTATTTCAAATGAGGTGTACGGAGACGTACCTTTGCTAGAGGAGATGGGGAAAAAGGTTAACTACCGCCCAGATTTTGGCCTTTCAT
TATTGGATGGAATGCTTTGCTATTATACTACTACTAATGATGGTTCATTGGGTTCTTTTAAGTTGTGGGCTATGAAAGACTATGGTGTCAAGGAATCT
TGGATAGTATTGTGTACCTTACAATTCAATGGTCTTGTTATAGCCAAACCACGATATAGGTTTCCAGATGGTGAGCTGCTACTATTTTCACCGGGGG
AATTCCCACATCAATGTGGGGTAGTTAGCTGTTTTACATTCCAGACATCCAAAGGAAAATTTGGATTTTGGCCTGAACGCGGAGGAACCTTTGAGA
AAGGAATCATTTATAGAGAAAGCTTGATTTCTCCAAAATTAGTTATCTAG
