# Supplementary material for: F-Box Family Genes, LTSF1 and LTSF2, Regulate Low-Temperature Stress Tolerance in Pepper (Capsicum chinense)
Source: Plants (Basel). 2020 Sep 11;9(9):1186. doi: 10.3390/plants9091186 (PMC7570372; doi:10.3390/plants9091186)
Supplement: Supplementary file 1 [file plants-09-01186-s001.zip › Table S2.pdf]

Table S2. qPCR primers used in this study.

Primer Name	Sequence	Reference
NtGST-F	AGCACCCTTACCTTTCCCTC	[52]
NtGST-R	GCAGCTTCTCCAATCCCTTAAC	
NtCAT-F	CACAGCCACGCTACTCAAGAC	[52]
NtCAT-R	CCACCCACCGACGAATAAAG	
NbAPX-F	GGAGTGGTTGCTGTTGAAGTC	[52]
NbAPX-F	GGAGAGCCTTGTCTGATGG	
NtSOD-F	CAACTCCACGGCTTCCAGAC	[52]
NtSOD-R	TGGGTCCTGATTAGCAGTGGT	
NbActin-F	CCAGGTATTGCTGATAGAATGAG	[52]
NbActin-R	CTGAGGGAAGCCAAGATAGAG	
LTSF1-RT-F	TCCAGATGGTGAGCTGCTACTATTTT	Present study
LTSF1 -RT-R	TGGATGTCTGGAATGTAAAACAGCTAAC	
LTSF2-RT-F	TCCAGATGGTGGGCTGCTACTATATG	Present study
LTSF2-RT-R	TGAATGTCTGGAATGTAAAACAGCTAGG	
CaActin-F	AGGAATGGTTAAGGCTGGATTT	Present study
CaActin-R	TCTTCTCCATATCGTCCCAGTT	
NbActin-F	CCAGGTATTGCTGATAGAATGAG	Present study
NbActin-R	CTGAGGGAAGCCAAGATAGAG	

Reference

52. Li, Y.; Zhang, L.; Wang, X.; Zhang, W.; Hao, L.; Chu, X.; Guo, X. Cotton GhMPK6a negatively regulates osmotic tolerance and bacterial infection in transgenic *Nicotiana benthamiana*, and plays a pivotal role in development. *FEBS J.* **2013**, *280*, 5128-5144.
